# Cloning of a new HSP70 gene from western flowerthrips, *Frankliniella occidentalis*, and expression patterns during thermal stress

**DOI:** 10.7717/peerj.7687

**Published:** 2019-09-25

**Authors:** Xiao-xiang Zhang, Jing Qin, Jia-Wen Yuan, Ming-Xing Lu, Yu-Zhou Du

**Affiliations:** 1 College of Horticulture and Plant Protection & Institute of Applied Entomology, Yangzhou University, Yangzhou, China; 2 Joint International Research Laboratory of Agriculture and Agri-Product Safety, The Ministry of Education, Yangzhou University, Yangzhou, China

**Keywords:** HSP70, *Frankliniella occidentalis*, Temperature, Recovery time, Expression pattern

## Abstract

*Frankliniella occidentalis* (Pergande) is an invasive pest that endangers a wide variety of horticultural and agronomic crops. HSP70 is the most important member of the heat shock protein (HSP) family and plays an important role in insect thermal tolerance. In this study, a new gene encoding HSP70 from *F. occidentalis*, *Fohsp706*, was selected from the *F. occidentalis* transcriptome exposed to thermal stress (40 °C) and cloned by RT-PCR and RACE. Further characterization indicated that *Fohsp706* localizes to the cytoplasm and does not contain introns. Quantitative real-time reverse transcriptase PCR indicated that *Fohsp706* expression was significantly up-regulated by thermal stress; furthermore, there were significant differences in *Fohsp706* expression in adults and second instar nymphs after heat stress. Our results indicated that *Fohsp706* contributes to thermotolerance in *F. occidentalis* and provides another example of how this pest adapts to unfavorable environmental conditions.

## Introduction

According to the Intergovernmental Panel on Climate Change (IPCC), the mean global surface temperature will be 0.3–0.7 °C higher in years 2016–2035 than 1986–2005 (IPCC, 2013). Over the past 55 years in China, the mean number of high-temperature days has increased by 28.4% (Wang et al., 2016). Environmental temperature determines geographic distribution and abundance of insect (Bowler & Terblanche, 2008). As poikilothermic animals, the growth, development, and reproduction of insects can be directly impacted by temperature, thus resulting in behavioral changes. Temperature can alter ecosystem stability and may result in recurrent insect outbreaks (Nelson, Bjørnstad & Yamanaka, 2013). Extreme temperatures can cause the destruction of insect cuticles, water loss, ion imbalances, and inactivation of proteins (Zhang, 2011).

Heat shock proteins (HSPs) are widely distributed in eukaryotic organisms. They are generally synthesized rapidly after temperature stress and help organisms adapt to adverse environments (Lindquist, 1986; Sǿrensen, Kristensen & Loeschcke, 2010; Sun et al., 2014). Heat shock proteins often function as molecular chaperones and facilitate the refolding of denatured proteins (Hartl, 1996); they are classified into HSP100, HSP90, HSP70, HSP60, HSP40, and small HSPs families according to homology and molecular weight (Lindquist & Craig, 1988; Morimoto, Tissieres & Georgopoulos, 1990; Kim, Kim & Kim, 1998). Among them, HSP90 and HSP40 are related to HSP70 in function. HSP90 chaperone activity requires collaboration with a subset of the many HSP90 cochaperones, including the HSP70 chaperone. In higher eukaryotes, the indirect collaboration between HSP90 and HSP70 involves Hop, a cochaperone interacting with both HSP90 and HSP70 (Kravats et al., 2018). HSP40 proteins contain the J domain which they bind to HSP70s. HSP40 proteins stimulate the ATPase activity of HSP70 and actually determine the activity of HSP70 by stabilizing their interaction with substrate proteins (Qiu et al., 2006).

The HSP70 family members are further subdivided into inducible (HSP70) and cognate forms (HSC70s). Inducible HSP70 genes have no introns or have relatively short introns and are preferentially translated; thus, HSP70s rapidly accumulate in response to adverse environmental stimuli (Gkouvitsas, Kontogiannatos & Kourti, 2009; Sǿrensen, 2010; Zhang & Denlinger, 2010). Cognate forms of HSP70 genes contain more introns, and intron numbers are conserved in vertebrates and are variable in invertebrates (Chuang, Ho & Song, 2007). HSP70s also have different localization signals; e.g. EEVD, HDEL, and PEAEYEEAKK for localization to the cytoplasm, endoplasmic reticulum, and mitochondria, respectively (Guy & Li, 1998).

*Frankliniella occidentalis* occurs worldwide and threatens both horticultural and agronomic crops. The pest has invaded many parts of China and exhibits a pattern of expansion from northern to southern regions (Zhang et al., 2003; Lv et al., 2011). The strong temperature tolerance and rapid domestication of *F. occidentalis* contribute to its fast, unrestricted dissemination in China. Studies have shown that antioxidant enzymes in *F. occidentalis* can effectively reduce the oxidative damage caused by high temperatures (Zheng, 2015). In addition, temperature tolerance in *F. occidentalis* is also conferred by inducible *hsp* genes including *Fohsp40, Fohsp60, Fohsp70*, and *Fohsp90* (Li et al., 2014; Lu et al., 2016; Qin et al., 2017).

The expression of *hsp* genes in *F. occidentalis* is related to the intensity and duration of stress. Differences in *hsp* expression were previously in *F. occidentalis* in response to high-temperature stress; for example, the expression of *Fohsp90* and *Fohsc70* reached a maximum at two hours, while *Fohsp60* reached maximal levels at six hours (Li, 2013). Genes encoding six forms of HSP70 were previously identified in *F. occidentalis* and differ in selected characteristics and responses to thermal stress (Lu et al., 2016; Qin et al., 2017; Qin et al., 2018). In this study, we isolate and analyze characteristic of *Fohsp70*, a new gene encoding an HSP70 form in *F. occidentalis*. Furthermore, we evaluated and compared *Fohsp70* expression during both high- and low-temperature stress and after different recovery times. The results provide a foundation for future studies on the mechanism of thermotolerance in *F. occidentalis*.

## Materials and Methods

### Insects

*Frankliniella occidentalis* adults were originally collected from Zhejiang Academy of Agricultural Sciences in September 2008 and the adults reared in the laboratory according to Li et al. (2011). The experimental colony was fed on *Phaseolus vulgaris* maintained in a QHX-300BS-III climate chamber at 25 ± 1 °C, 70–80% RH, and a 16:8 h light: dark photoperiod.

### High and low temperature treatments

Second instar larvae (*n* = 120) were collected, placed in glass tubes and exposed to various temperatures for 1 h. Larvae were exposed to cold (−6, −8, −10, −12, −14 °C) and hot (33, 35, 37, 39, 41 °C) conditions using a temperature controller (DC-3010, Ningbo, Zhejiang, China). The control group consisted of thrips maintained at 26 °C, and all treatments were replicated four times.

### Recovery times

Adults (*n* = 200) were collected and placed together in glass tubes; two replicates of each sample were prepared. The adults were exposed to 40 °C for 1 h in a constant temperature water bath and allowed to recover at 26 °C for 0, 1, 1.5, 2 and 2.5 h. Treated and control samples were frozen in liquid nitrogen for 5 min and then stored at −80 °C. Each recovery period was replicated four times. The same protocol was used for second instar nymphs and pupae.

### RNA extraction and cDNA synthesis

Total RNA was extracted from *F. occidentalis* adults using the SV Total RNA Isolation System (Promega, San Luis Obispo, CA, USA). The concentration and quality of RNA were analyzed by spectrophotometry (Eppendorf Bio Photometer Plus, Hamburg, Germany) and agarose gel electrophoresis. Total RNA (1 μg) was used as a template and oligo(dT)_18_ primers were used to generate the first strand cDNA as recommended in the First Strand cDNA Synthesis Kit (Clontech, Mountain View, CA, USA).

### Cloning full-length *Fohsp706*

Primers (Table 1) were designed to amplify DNA fragments of *F. occidentalis* based on sequences obtained from the transcriptome. PCR reactions were as follows: 94 °C for 3 min, 19 cycles of 94 °C for 30 s, 64–44 °C (decreasing by 1 °C/cycle) for 30 s, 72 °C for 1 min, and then 30 cycles of 94 °C for 30 s, 45 °C for 30 s, and 72 °C for 1 min, with extension at 72 °C for 10 min. Purified products were cloned into the pGEM-T Easy vector (Promega, Madison, WI, USA) and transformed into competent *Escherichia coli* DH5α cells for sequencing.

**Table 1:** Primers in this study.

Primer name	Primer sequences (5′-3′)	Purpose
*hsp706* DP-F *hsp706* DP-R	GCTTGATTGGCAGACGATTTGAG CAAGTGAAAGAGGGGTAACATCC	Amplification of internal fragment
*hsp706* RACE-5′-1 *hsp706* RACE-5′-2	GTGAACTAAGTCTCAATCTC TGGTTGTTGTTGGTATAAGAAGACGA	5′RACE
*hsp706* RACE-3′-1 *hsp706* RACE-3′-2	ATACACCAGAATCTCACTTG TGTGCTCCGAGGACTTTATTTCAGGG	3′RACE
*hsp706* cDNA-F *hsp706* cDNA-R	AGCAGGCTGGCAGGCACAAC GGGACTGGTAACAGGAGCCG	Verification of full-length cDNA
*hsp706* DNA-F *hsp706* DNA-R	AGCAGGCTGGCAGGCACAAC GGGACTGGTAACAGGAGCCG	Verification of genome
*hsp706* RT-F *hsp706* RT-R	CTTTAGCGGCGACAGTTGGA GGAGCACAAACCGTGACCAA	Real-time quantitative PCR

Gene-specific primers (Table 1) were designed to obtain 5′ and 3′ regions using the SMART RACE cDNA Amplification Kit (Clontech, Mountain View, CA, USA) based on the sequence of partial fragments. PCR parameters were as follows: 94 °C for 3 min, 35 cycles of 94 °C for 30 s, 68 °C for 30 s, and 72 °C for 3 min, followed by extension at 72 °C for 10 min. Bands of the expected size were cloned and sequenced as described above.

### Sequence analysis of *Fohsp706*

Nucleotide and amino acid sequence similarities were evaluated using the BLAST program available at NCBI (https://blast.ncbi.nlm.nih.gov/Blast.cgi). ORF Finder (https://www.ncbi.nlm.nih.gov/orffinder/) was used to identify complete open reading frame. Amino acid sequences (Gasteiger et al., 2003) were deduced using ExPASy sequence analysis tools, and motifs were identified using ScanProsite software (http://www.expasy.org/tools/scanprosite). Homology searches were carried out using Blast programs of NCBI (https://blast.ncbi.nlm.nih.gov/Blast.cgi). A phylogenetic tree of insect HSP70s was constructed by neighboring joining, minimum evolution, maximum likelihood, and maximum reduction methods using MEGA X (Kumar et al., 2018).

### Amplification of genomic DNA

Genomic DNA of *F. occidentalis* adults was extracted according to AxyPrep instructions, and samples were stored at −20 °C. Based on the full-length cDNA sequence of *Fohsp706* in *F. occidentalis*, multiple primers (Table 1) were designed to amplify the genomic sequence. PCR products were cloned and sequenced as described above. Homologous sequences were obtained using BlastN. After confirmation, full-length *hsp70* genomic sequences and the DNA sequence of the new *hsp70* gene (*Fohsp706*) were compared, and intron distribution was analyzed.

### Quantitative real-time reverse transcriptase PCR (qRT-PCR)

Real-time quantitative cDNA was synthesized using instructions provided with the PrimeScript RT Reagent Kit (Bio-Rad, Berkeley, CA, USA), and primers were designed using Primer 5.0 software (Table 1). Reactions were conducted using a CFX-96 Real-Time PCR System (Bio-Rad, Berkeley, CA, USA), and each PCR reaction included three replicates. PCR reactions included iTaq Universal SYBR Green Supermix (2×) (Bio-Rad, Berkeley, CA, USA), one μL of each forward and reverse primer (10 μmol L^–1^) (Table 1), two μL of cDNA template (2.5 × 10^–4^ μg μL^–1^), and six μL of ddH_2_O. Quantitative real-time reverse transcriptase PCR conditions were as follows: 95 °C for 30 s, 40 cycles of 95 °C for 30 s, and 56.3 °C for 15 s; melting curve analysis was then performed to determine the specificity of PCR products.

### Statistical analysis

Expression levels in each treatment were analyzed using the 2^–ΔΔCt^ method (Livak & Schmittgen, 2001) and differences were detected using Tukey’s multiple comparison method. All data were analyzed and processed using SPSS16.0 software, and the significance level was *P* < 0.05.

## Results

### Characterization of a new *hsp70* in *F. occidentalis*

A 257-bp fragment was amplified by PCR with internal primers (hsp706DP-F/R, Table 1) using *F. occidentalis* cDNA as a template and then cloned and sequenced. BlastN alignment revealed 75–91% identity with *hsp70* in other insects, suggesting that the fragment encoded part of an *hsp70* gene in *F. occidentalis*. The 5′-terminal (563 bp) and 3′-terminal (1519 bp) fragments of the putative *F. occidentalis hsp70* were subsequently amplified by RACE. The full-length *hsp70* was then obtained by splicing the 257-, 563-, and 1519-bp fragments together with DNAMAN (version 6.0, Lynnon Biosoft, America). The full-length sequence was verified by the cDNA sequence and was deposited in GenBank as *Fohsp706* (accession no. MK603518).

The ORF Finder revealed that *Fohsp706* contained a 5′ untranslated region (159 bp 5′ UTR), a 1917-bp ORF and 3′ untranslated region (1713 bp 3′UTR); the 3′UTR contained a typical polyA tail. ProtParam analysis tool (ExPASy) showed that *Fohsp706* encoded 638 amino acids with a theoretical molecular weight of 70.1 kDa, a formula of C_3051_H_4912_N_876_O_981_S_16_ and an isoelectric point of 5.31. Alanine (Ala) was the most prevalent amino acid (9.6% of total), followed by aspartic acid (Asp), which accounted for 7.7%. The predicted protein contained 94 negatively charged residues (Asp + Glu) and 81 positively charged residues (Arg + Lys). The overall stability index of the protein is 40.51 (≤40 is considered stable), the aliphatic index is 81.66, and the hydrophilicity index is −0.461 (non-hydrophilic). ScanProsite showed that the deduced *Fo*HSP706 amino acid sequence contained three HSP70 signature sequences: IDLGTTYS (6–13 aa), IFDLGGGTFDVSVL (194–207 aa) and VVLVGGSTRIPKVQS (332–346 aa). The C-terminal end is a typical EEVD (Glu-Glu-Val-Asp), indicating that the protein exists in the cytoplasm of *F. occidentalis* (Fig. S1).

### Genome structure of *Fohsp706*

**Figure 1:**
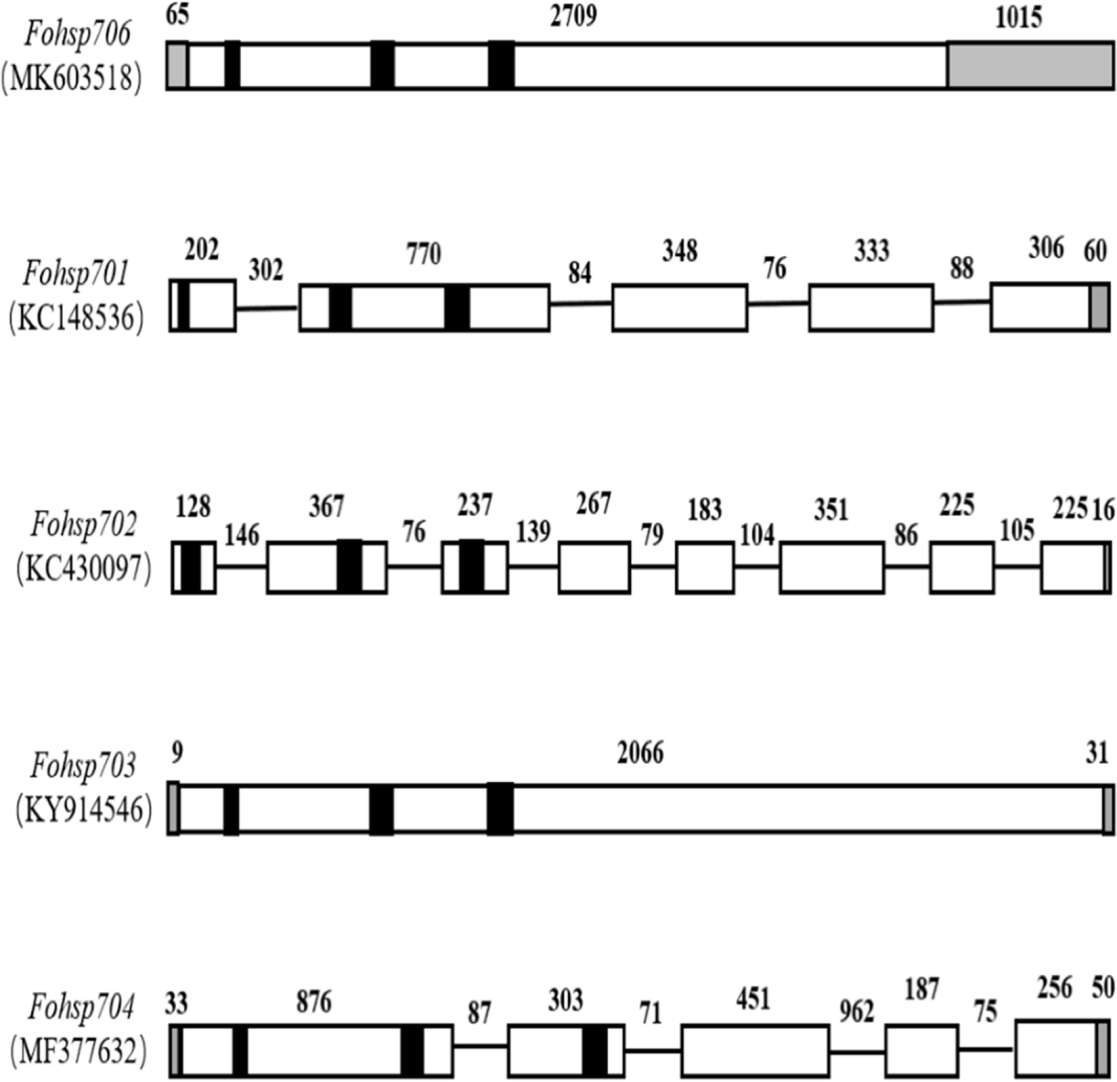
Genomic forms of *Fohsp70*s in *F. occidentalis*. Grey boxes represent non-coding regions, black boxes represent signatural sequences of HSP70 family, blank boxes represent exons, straight lines represent introns, numbers indicate the lengths of exons, introns and non-coding regions.

A pair of primers, *hsp706*DNA-F/R (Table 1.) was designed to amplify the genomic copy of *Fohsp706*, and a 2022-bp sequence was obtained. In Table 2, the genomic structures of *Fohsps* from *F. occidentalis* were compared, except *Fohsc705*, the genomic structure of which was not available. The genomic form of *Fohsp706* had no introns, which suggested that the gene might be inducible. In contrast, *Fohsp701* (accession no. KC148536), *Fohsp702* (accession no. KC430097), and *Fohsp704* (accession no. MF377632) have 4, 7, and 4 introns, respectively; whereas, *Fohsp703* (accession no. KY914546) has no introns. Therefore, this indicates that not only the number of introns, but also the distribution positions of introns in HSP70 gene of the same species may be different (Fig. 1).

**Table 2:** Characteristics of six *hsp70*s in *F. occidentalis*.

Genes	MW (kDa)	Number of introns	Response to heat stress	Response to cold stress
*Fohsc701*	69.81	4	N	N
*Fohsc702*	72.93	7	N	N
*Fohsp703*	73.6	0	M	M
*Fohsc704*	75.0	4	N	N
*Fohsc705*	54.5	6	M	M
*Fohsp706*	70.1	0	H	M

**Notes:**

H, highly induced; M, moderately induced; N, not induced; and ND, not determined.

### Phylogenetic analysis of *Fo*HSP706

**Figure 2:**
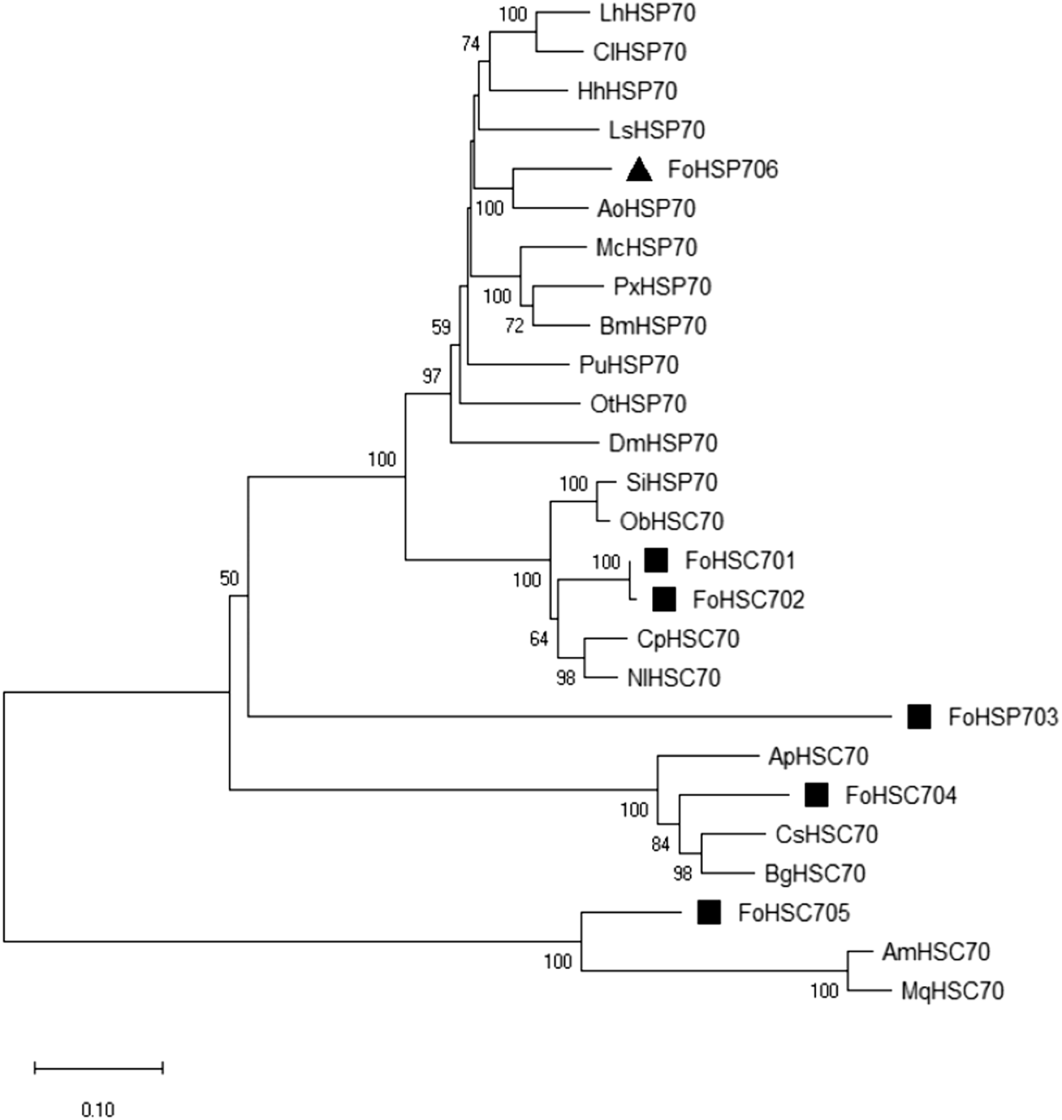
Phylogenetic tree of HSP70s from multiple insects based on the maximum likelihood method. *FoHsp70* (this study) is marked with a solid square. HSP70 proteins and GenBank accession numbers are as follows: FoHSP706 (MK603518), FoHSC701 (JX002706.1), FoHSC702 (KC148536.1), FoHSP703 (KY986660.1), FoHSC704 (KY914547.1), FoHSC705 (MF377632.1), *Bombyx mori* BmHSP70 (AB035326.1), *Plutella xylostella* PxHSP70 (AB325801.1), *Drosophila melanogaster* DmHSP70 (AH007395.2), *Anaphothrips obscurus* (AXB26576.1), *Halyomorpha halys* (XP_024214745.1), *Paratlanticus ussuriensis* (AFP54305.1), *Laodelphax striatella* (AQP31338.1), *Papilio polytes* (XP_013148831.1), *Onthophagus taurus* (XP_022912059.1), *Melitaea cinxia* (AGR84224.1), *Plutella xylostella* (ADK94699.1), *Lygus hesperus* (AFX84560.1), *Cyrtorhinus lividipennis* (AXU24955.1), *Solenopsis invicta* (XP_025995000.1), *Chrysopa phyllochroma* (AHY95944.1), *Nilaparvata lugens* (ADE34170.1), *Ooceraea biroi* (XP_011348101.1), *Cryptotermes secundus* (XP_023710212.1), *Blattella germanica*, (PSN32841.1), *Agrilus planipennis* (XP_025835266.1), *Apis mellifera* (XP_623199.2), and *Melipona quadrifasciata* (KOX73871.1). Numbers on branches represent bootstrap values obtained from 1,000 replicates.

ClustalW (Thompson, Gibson & Higgins, 2002) indicated that the deduced amino acid sequence of *Fo*HSP706 was over 70% similar to other HSP70s (Fig. S2). Several HSP70 amino acid sequences, including the *Fo*HSP703 mentioned above, were compared with *Fo*HSP706. Phylogenetic trees of HSP70s were constructed using MEGA X and the neighbor joining, minimum evolution, maximum reduction, and maximum likelihood methods (Zuckerkandl & Pauling, 1965; Kumar et al., 2018). The tree was divided into five branches, and these six *Fo*HSP70s were distributed in different branches (Fig. 2). Phylogenetic analysis showed that *Fo*HSP706 has a close evolutionary relationship with HSP70 of *Anaphothrips obscurus* (Muller), which also belongs to Thripinae (Gao & Feng, 2018).

### Expression of *Fohsp706* in response to cold and heat shock

**Figure 3:**
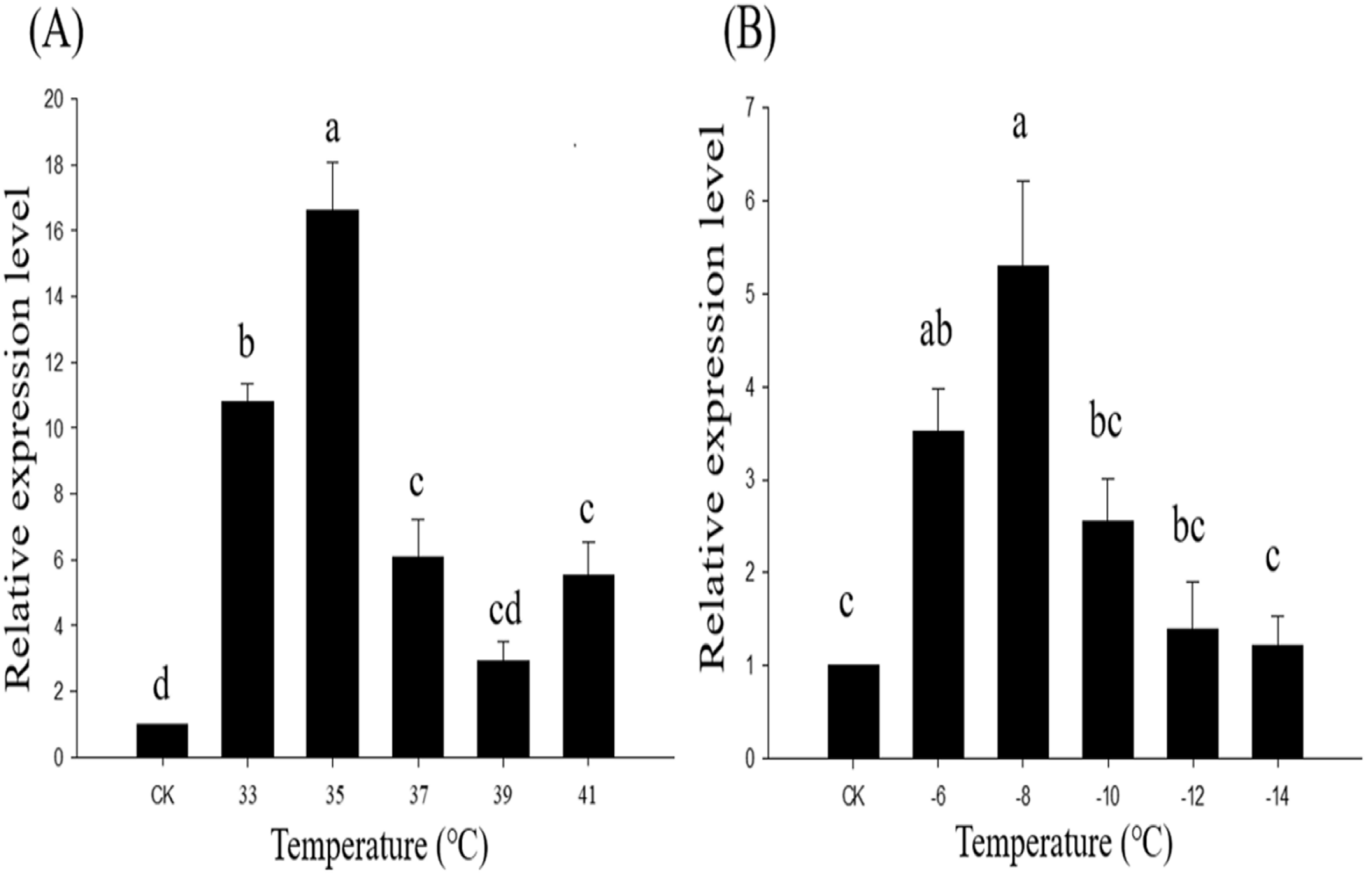
Expression levels of *Fohsp706* during a 2-hexposure to *Frankliniella occidentalis* larvae. (A) *Fohsp706* expression in response to heat stress at 33, 35, 37, 39, 41 °C (B). Expression in response to cold stress at −6, −8, −10, −12, and −14 °C. Expression levels were normalized with respect to *RPL32 for heat stress* and *18S rRNA for cold stress*, and histograms indicate relative expression levels. All statistics indicate means ± SE. Columns labeled with different letters indicate significant differences using a one-way ANOVA followed by Tukey’s multiple comparison analysis. All statistics are presented as means ± SE.

RT-PCR was used to study expression profiles of *Fohsp706* in second instar larvae of *F. occidentalis*. qRT-PCR assays resulted in the production of single amplicons with efficiency values between 93.5 and 107.3% and an *R*^2^ value of 0.980, which meets the basic requirements of real-time quantitative research. The relative mRNA levels of *Fohsp706* were compared at −14, −12, −10, −8, −6, 26, 33, 35, 37, 39 and 41 °C. *Fohsp706* was significantly induced in response to hot and cold temperatures as compared with the control (26°C). With respect to high-temperature stress, expression of *Fohsp706* was highest at 35 °C (Fig. 3A); in response to cold stress, expression of *Fohsp706* was highest at −8 °C (Fig. 3B).

**Figure 4:**
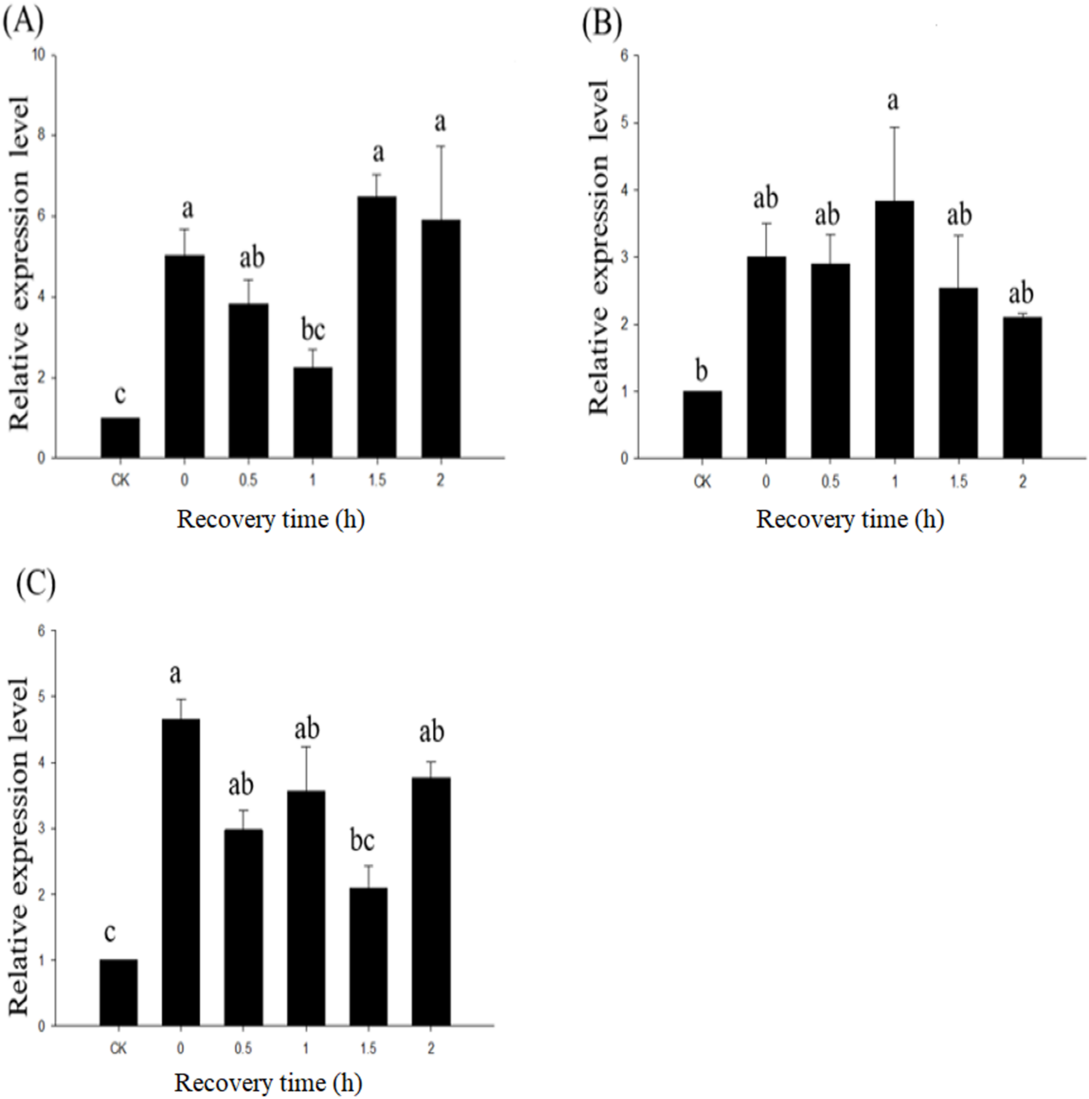
Relative expression of *Fohsp706* in *F. occidentalis* at 0, 0.5, 1, 1.5 and 2 h recovery times at 26 °C after exposure to heatshock at 40 °C. Relative expression of *Fohsp706* in *F. occidentalis* adults (A), pupae (B), and (C) larvae. CK: expression level at 25 °C. All statistics are presented as means ± SE. Columns labeled with different letters indicate significant differences using one-way ANOVA followed by Tukey’s multiple comparison analysis.

In response to high-temperature stress (40 °C), the expression of *Fohsp706* in *F. occidentalis* adults after recovery times of 0, 0.5, 1.5, and 2 h remained significantly induced in comparison to the control group. However, the recovery time of 1.0 h was not significantly different from the control group (Fig. 4A). Statistical analysis showed that different recovery times after high-temperature exposure had no effect on *Fohsp706* expression in *F. occidentalis* adults (*F*_5,__17_ = 9.529, *P* < 0.001). Contrary to adults, *Fohsp706* expression in pupae was significantly induced after the recovery time of 1 h, but not at other time intervals (Fig. 4B). *Fohsp706* expression in larvae showed significant induction after recovery times of 0, 0.5, 1, and 2 h (but not 1.5 h) as compared to the control (Fig. 4C). Thus, different recovery times after high-temperature treatment impact *Fohsp706* expression in *F. occidentalis* pupae and larvae.

## Discussion

HSP70 family members often contain motifs for subcellular localization; for example, the seven *hsp70* genes encode different signature sequences that target the protein products to different subcellular locations. In this study, the C-terminal motif in *Fo*HSP706 contains EEVD, indicating that the protein localizes to the cytoplasm. Genes encoding five forms of HSP70 were previously identified in *F. occidentalis* which are separately named *Fohsc701*, *Fohsc702*, *Fohsp703, Fohsc704, Fohsc705*, thus, the new *hsp70* was named *Fohsp706*. Some HSP70s contain KDEL at the C-terminus, which targets the protein to the endoplasmic reticulum. Most members of the HSP70 family in *F. occidentalis* end with EEVD and KDEL (Lu et al., 2016). Recently, Qin et al. (2017) described *Fo*HSP703, a heat-induced HSP70 protein that contains a unique APAA motif at its C-terminus, which is distinct from previously described insect forms of HSP70.

In this study, multiple sequence alignment and phylogenetic tree construction identified *Fohsp706* as a member of the HSP70 family. The phylogenetic tree showed the decentralized distribution of *Fo*HSP70s, indicating that HSP70s in insecta are diverse and mutated. The *Fo*HSP706 in this study was not located in the same branch with other *Fo*HSP70s, but they were close to Hemiptera. The result suggested that the evolutionary history of *Fo*HSP706 be parallel to insects in Hemiptera. *Fohsp706* did not contain introns and was induced by high and low temperature stress; thus, it resembles with *Fohsp703* (Table 2). In *F. occidentalis* larvae, the expression of *Fohsp706* in this study peaked at 35 °C. According to Qin (2018), all *F. occidentalis* larvae survived after 1-h exposure at 35 °C. This might be related to the expression of *Fohsp706* which also peaked at 35 °C. After 1-h exposure at cold stress in the range of −8 °C to −14 °C, the expression level of *Fohsp706* decreased with the decrease of temperature showing a negative correlation to the mortality of *F. occidentalis* larvae after cold stress.

Interestingly, we discovered that intron numbers and positions were quite different in *Fohsps.* *Fohsc701*, *Fohsc702*, *Fohsc704*, and *Fohsc705* contain introns (Table 2); however, only *Fohsc705* was induced by temperature (Lu et al., 2016, Qin et al., 2018). It supported that highly expressed genes either lack introns or have relatively short introns relative to weakly expressed genes (Castillo-Davis et al., 2002). Thus, distinct differences exist among *hsp70* genes within *F. occidentalis* and intra-specific variation can lead to differences in genomic structure and function. Besides, Wang et al. (2014) also did some research about responses of HSP70 genes from *F. occidentalis* to thermal stress and FoHSP70 was strongly induced by both heat and cold stress in their study, but no information of genome structure was involved. We presume that *F. occidentalis* HSP70 in their research may lack introns with such high expression level in response to heat stress, whereas this is only our assumption.

Numerous reports have shown that HSP70 plays an important role in insect tolerance to thermal stress (Huang & Kang, 2007; Udaka, Ueda & Goto, 2010; Lu et al., 2016). In this study, we investigated whether *Fohsp70*6 expression in adult, larval, and pupal forms of *F. occidentalis* was impacted by different recovery times after high-temperature stress (40 °C). Our results indicated that *Fohsp706* was differentially expressed in different developmental stages. In *F. occidentalis* adults, *Fohsp706* expression peaked at the 1.5-h recovery time point. However, *Fohsp706* expression reached a maximal level at the 0-h time point in larvae. In pupae, *Fohsp706* expression was significantly induced at the 1-h time point but no significant differences were observed among different recovery times. To some extent, these results reflect the short-term nature of HSP70, which has been observed for some plant forms of the protein. Plant HSPs accumulated within 3–5 min after heat shock and reached maximal levels at 1–2 h; protein levels were significantly reduced at 6 h and undetectable at 12 h (Kimpel et al., 1990). In *Drosophila melanogaster* exposed to 0 °C, *hsp70Aa* expression peaked at the 2-h recovery time point (25 °C), and then gradually decreased (Colinet, Lee & Hoffmann, 2010).

*Frankliniella occidentalis* is an important pest on vegetables and horticultural crops worldwide. It can spread quickly and cause severe damage due to its small size, diverse modes of reproduction, rapid multiplication, and resistance to pesticides. In this study, we investigate the impact of thermal stress on *Fo*HSP706, a new form of HSP70 in *F. occidentalis*. Additional studies on the HSP70 family in *F. occidentalis* are warranted and will hopefully provide a theoretical basis for prevention and control of this invasive pest.

## Conclusion

*Frankliniella occidentalis* is an important pest on vegetables and horticultural crops worldwide. It can spread quickly and cause severe damage due to its small size, diverse modes of reproduction, rapid multiplication, and resistance to pesticides. In this study, we investigate the impact of thermal stress on *Fo*HSP706, a new form of HSP70 in *F. occidentalis*. Additional studies on the HSP70 family in *F. occidentalis* are warranted and will hopefully provide a theoretical basis for prevention and control of this invasive pest.

## Supplemental Information

10.7717/peerj.7687/supp-1 Supplemental Information 1 Nucleotide and deduced amino acid sequences of *Fo*HSP706.

10.7717/peerj.7687/supp-2 Supplemental Information 2 Multiple sequence alignment of deduced amino acids from *Fo*HSP706 with analogous proteins from other species.

10.7717/peerj.7687/supp-3 Supplemental Information 3 Fohsp706 qRT-pcr data. A represents Adults, L represents larvae, P represents pupae.

## Additional Information and Declarations

### DNA Deposition

The following information was supplied regarding the deposition of DNA sequences: The Fohsp706 sequence is accessible at GenBank: MK603518.

### Data Availability

The following information was supplied regarding data availability: The raw data are available in a Supplemental File.
