# Supplementary material for: Cloning of a new HSP70 gene from western flowerthrips, Frankliniella occidentalis, and expression patterns during thermal stress
Source: PeerJ. 2019 Sep 25;7:e7687. doi: 10.7717/peerj.7687 (PMC6765361; doi:10.7717/peerj.7687)
Supplement: Supplemental Information 1 [file peerj-07-7687-s001.pdf]

```
-159    ACATGGGGAGTTTTGATACAGCGATCACAGCGACCAGACGCAAGCGACAACGCGAAGTCTAGCGACTTTCTCTGT
-84     GCGTGAACGTGAACGCGAGTTTCTAAGTGCGAAGTGCAGTTTTTCTCCGAGTTCAGTGAACTAAGTCTCAATCTC
-9      AGCACCAAG
1       ATGCCAGCCGTGGGAATCGACTTGGGCACCACCTACTCCTGCGTGGGCGTGTGGCAGCAGGGCAAGGTGGAGATC
1        M  P  A  V  G  I  D  L  G  T  T  Y  S  C  V  G  V  W  Q  Q  G  K  V  E  I
76      ATCGCCAACGACCAGGGCAACCGCACCACCCCCAGCTATGTGGCCTTCACCGACACCGAGAGGCTGATCGGCGAC
26       I  A  N  D  Q  G  N  R  T  T  P  S  Y  V  A  F  T  D  T  E  R  L  I  G  D
151     GCGGCCAAGAACCAGACCGCCATGAACCCCAAGGACACCGTGTTCGACGCCAAGCGACTCATCGGGCGGCGCTTC
51       A  A  K  N  Q  T  A  M  N  P  K  D  T  V  F  D  A  K  R  L  I  G  R  R  F
226     GACGACCCCAAGATCCAGGCCGACATCAAGCAGTGGCCCTTCAGCGTCATCAGCGACAGCGGCAAGCCCAAGATC
76       D  D  P  K  I  Q  A  D  I  K  Q  W  P  F  S  V  I  S  D  S  G  K  P  K  I
301     CAGGTCACGTTCAAGGGCGAGCGCAAGGTGTTCTCCCCGGAGGAGATCAGCTCCATGGTGTTGACGAAGATGAAG
101      Q  V  T  F  K  G  E  R  K  V  F  S  P  E  E  I  S  S  M  V  L  T  K  M  K
376     GAGACGGCCGAGGCGTACCTGGGCAGCACGGTCCGGGACGCGGTGGTCACCGTGCCCGCCTACTTCAACGACAGC
126      E  T  A  E  A  Y  L  G  S  T  V  R  D  A  V  V  T  V  P  A  Y  F  N  D  S
451     CAGCGCCAGGCCACCAAGGACGCGGGCGTCATCGCGGGGCTCAACGTCATGAGGATCATCAACGAGCCCACGGCC
151      Q  R  Q  A  T  K  D  A  G  V  I  A  G  L  N  V  M  R  I  I  N  E  P  T  A
526     GCGGCGTTGGCCTACGGCCTGGACAAGAACCTCAAGGGCGAGCGCAACGTGCTCATCTTCGACCTGGGCGGCGGC
176      A  A  L  A  Y  G  L  D  K  N  L  K  G  E  R  N  V  L  I  F  D  L  G  G  G
601     ACCTTCGACGTGTCGGTGCTCTCCATCGACGAGGGCTCCCTGTTCGAGGTCAAGTCGACGGCCGGAGACACGCAC
201      T  F  D  V  S  V  L  S  I  D  E  G  S  L  F  E  V  K  S  T  A  G  D  T  H
676     CTGGGCGGCGAGGACTTTGACAGCCGGCTCGTCAACCACCTGGCCGAGGAGTTCAAGCGCAAATACAAGAAGGAC
226      L  G  G  E  D  F  D  S  R  L  V  N  H  L  A  E  E  F  K  R  K  Y  K  K  D
751     GTGACGAGCAACCCCCGCGCCCTGCGTCGCCTGCGCACCGCGGCGGAGCGCGCCAAGCGCACGCTGTCGTCCAGC
251      V  T  S  N  P  R  A  L  R  R  L  R  T  A  A  E  R  A  K  R  T  L  S  S  S
826     ACCGAGGCCAACATCGAGATCGACGCGCTGTACGAGGGCATCGACTTCTACACCAAGGTGTCCCGGGCCCGCTTC
276      T  E  A  N  I  E  I  D  A  L  Y  E  G  I  D  F  Y  T  K  V  S  R  A  R  F
901     GAGGAGCTGTGCGCCGATCTGTTCCGCGCCACGCTCACCCCGGTCGAGCGCGCGCTGCAGGACGCCAAGATGAGC
301      E  E  L  C  A  D  L  F  R  A  T  L  T  P  V  E  R  A  L  Q  D  A  K  M  S
976     AAGAGCGACATCCACGACGTGGTCCTGGTGGGCGGCTCCACGCGCATCCCCAAGGTCCAGAGCCTGCTGCAGAAC
326      K  S  D  I  H  D  V  V  L  V  G  G  S  T  R  I  P  K  V  Q  S  L  L  Q  N
1051    TTCTTCTGCGGCAAGTCCCTCAACCTGTCCATCAACCCGGACGAGGCCGTGGCGTACGGCGCGGCCGTGCAGGCC
351      F  F  C  G  K  S  L  N  L  S  I  N  P  D  E  A  V  A  Y  G  A  A  V  Q  A
1126    GCCATCCTGTCCGGCGACCAGAGCGAGGCCATCCAGGACGTGCTGCTGGTGGACGTGGCGCCGCTGTCGCTCGGC
376      A  I  L  S  G  D  Q  S  E  A  I  Q  D  V  L  L  V  D  V  A  P  L  S  L  G
1201    ATCGAGACGGCGGGCGGCGTCATGACCAAGATCATCGAGCGCAACAGCCGCATCCCGTGCAAGCAGACGCAGACC
401      I  E  T  A  G  G  V  M  T  K  I  I  E  R  N  S  R  I  P  C  K  Q  T  Q  T
1276    TTCTCGACGTACGCCGACAACCAGCCGGCCGTCACCATCCAGGTGTTCGAGGGCGAGCGCGCCATGACCAAGGAC
426      F  S  T  Y  A  D  N  Q  P  A  V  T  I  Q  V  F  E  G  E  R  A  M  T  K  D
1351    AACAACCGCCTGGGCACCTTCGACCTGACGGGCATCCCGCCCGCCCCTCGCGGCGTCCCCAAGATCGAGGTCACC
451      N  N  R  L  G  T  F  D  L  T  G  I  P  P  A  P  R  G  V  P  K  I  E  V  T
1426    TTTGACCTGGACGCCAACGGCATCCTCAACGTCGCCGCCAAGGACACGAGCTCGGGCCGCTCCCAGAACATCACC
476      F  D  L  D  A  N  G  I  L  N  V  A  A  K  D  T  S  S  G  R  S  Q  N  I  T
1501    ATCAAGAACGACAAGGGCCGCCTCTCGGCCGACGAGATCCAGCGCATGGTGGACGAGGCGGAGCGGTACAAGGCC
501      I  K  N  D  K  G  R  L  S  A  D  E  I  Q  R  M  V  D  E  A  E  R  Y  K  A
1576    GAGGACGACCAGCAGCGCGACCGCGTCGCAGCGCGCAACCAGCTGGAGGGCTACGTGTTCAGCATCAAGCAGGCC
526      E  D  D  Q  Q  R  D  R  V  A  A  R  N  Q  L  E  G  Y  V  F  S  I  K  Q  A
1651    ATCGACGAGGCCGGCGACAAGCTCCCGGAGCAGGACAAGACCAGGGCGCGCGAGGAGTGCGAGGCGACGCTGCGC
551      I  D  E  A  G  D  K  L  P  E  Q  D  K  T  R  A  R  E  E  C  E  A  T  L  R
1726    TGGCTGGACAGCAACACCCTGGCCGACAAGGAGGAGTACGAGCACCGCCTCAAGGAGCTGCAGCAGAAGGTGCAG
576      W  L  D  S  N  T  L  A  D  K  E  E  Y  E  H  R  L  K  E  L  Q  Q  K  V  Q
1801    GAGGTCATGGTGCGCCTGCACTCGGGCGGCCCGGGCGCGTCACAGCCCCAGGCACCCCAGGGCTGCGGGCAGCAG
601      E  V  M  V  R  L  H  S  G  G  P  G  A  S  Q  P  Q  A  P  Q  G  C  G  Q  Q
1876    GCTGGCAGGCACAACGGCCCCACCGTCGAGGAGGTCGACTAAACCAACCCAGTCATTAGTGTGATAAAGACTTTA
626      A  G  R  H  N  G  P  T  V  E  E  V  D  *
1951    CTACAAGTGAGATTCTGGTGTATATTTCTGTGTGAACATGATTGTTAGTTGCAGTATTTCTTAAATTCCAAAGAC
2026    GTGTGTAAGTGTGTATCTATATTTAAGTTTAGGTTGAATGTTATGTGTTTACATAATTTATTGGTTTCTGCATTA
2101    GCAGTGTGCCATGTTGACTGATTAATTGTTAATTTAAGTTTAGATAGCAAATGTTTTTCTTGTATGAATTTGATC
2176    TCTTAATCTTGTTTCCTGTTGAATTGTAGTACATTGCAGGGATGTAAACTGTGTACGTGAATTATTTAAAGCAAT
2251    AAAAAATTAGGTGATATTTTTTTAAACCTAAGTGTTGTATTTCCCCTTTCGTCTTCTTATACCAACAACAACCAA
2326    GATCAAAAAACTATTTTCTTCAAATCAGTGTACATACACAGAAAGTTTTACCTTTGAACTCTTGACTGCATTTTC
2401    TTTTACCAAAGCATTTTCACACTGTCAACAATAATTGAAAGCAAACCACTGTTTTACACGGAAATAACACTAACA
2476    GTTTTATTACTCACCCTATGAGCCAGGTATTGAATACAACATCACCCCCACTTTTTAACGCAATATTGATAGACC
2551    TTGTTTTGCTTTTTTTGTATTGAATAACACAAGAGAATGGGGAGAAGTAAAGATACAAATTACAAGGTAGGAAGGC
2626    TCAAATATTTGCTTGTCATAACATTTATCCAAATTAAAATATTTTTGTTTTGCAGTATTTGCTTTTTACGAACAT
2701    TACAGAATTTAAAGGACCCATAAAAGGAGCAGTGAACTGAGTTCGGTGTGAGGCCTTTTCTCAAGGGCCTAATCT
2776    GACACCATCAAGCATGTTTCAAATGTGTCGCGATGTTCGCGCGCGCCGACAGCAGCGCTTTAGCGGCGACAGTTG
```

```
2851     GAAATCTCCACCTCCACTTAGCAGACAGTCAAACCTAACTTCCCCTTTACTTTTGTTCTCAGCGTTATGAGTACA
2926     TTCAGACTTAATATGTTTATGTTCTTCAATTTTTATTGGTCACGGTTTGTGCTCCGAGGACTTTATTTCAGGGAC
3001     ATGAAACAGACATAAATGACTGATCATGAGCAGTACAAAATAAAAAATTTCTTGAACAGCCCTAGAACTTCAGAA
3076     CTTCAGGAACTTTGGACCCGAAAACTTCTGCATCAACGCTAAACCATGAGAAAATTAAATTTTGTTATGTTAATC
3151     GGGGAAACTGAAAGGGAAACACACAGTTTCCTCGTTTGACCCTCCTCTACTCAAGTTGTTCTCTTTCTTAGAAGG
3226     CGTGTGCTCGATTCTTCCATGCATCGTCGAAAGATCGGCTCCTGTTACCAGTCCCCTAACAGATGTACGTCTCCT
3301     GAGAACTTGTAAACAAACAAAGACGCAACGAAACTCGTCAGGGGAGGGCTTTGTCCTCAAGTTTGAAGTTTTGTA
3376     TGCTACTTGTCGAATCTACATTTATAGAAAATTTACTTTTAACTCAGCTTACCATTGAACAGACGACATGCATAA
3451     ACGAAGAAGTAGAGACAAGAATGACGTACAACCATCTGACACCAACATGATGTGCGTGGTTCCGAAGCAAAACGT
3526     AGAAAGTAAGCCGTCTAACCTAACTATCAGATTGTCATTAGTGTTGAGTTTAATAAAAATGGAAATTTTCTACCC
3601     ATAAAAAAAAAAAAAAAAAAAAAAAAAAAA
```
