# Supplementary figures and images for: Cloning of a new HSP70 gene from western flowerthrips, Frankliniella occidentalis, and expression patterns during thermal stress
Source: PeerJ. 2019 Sep 25;7:e7687. doi: 10.7717/peerj.7687 (PMC6765361; doi:10.7717/peerj.7687)

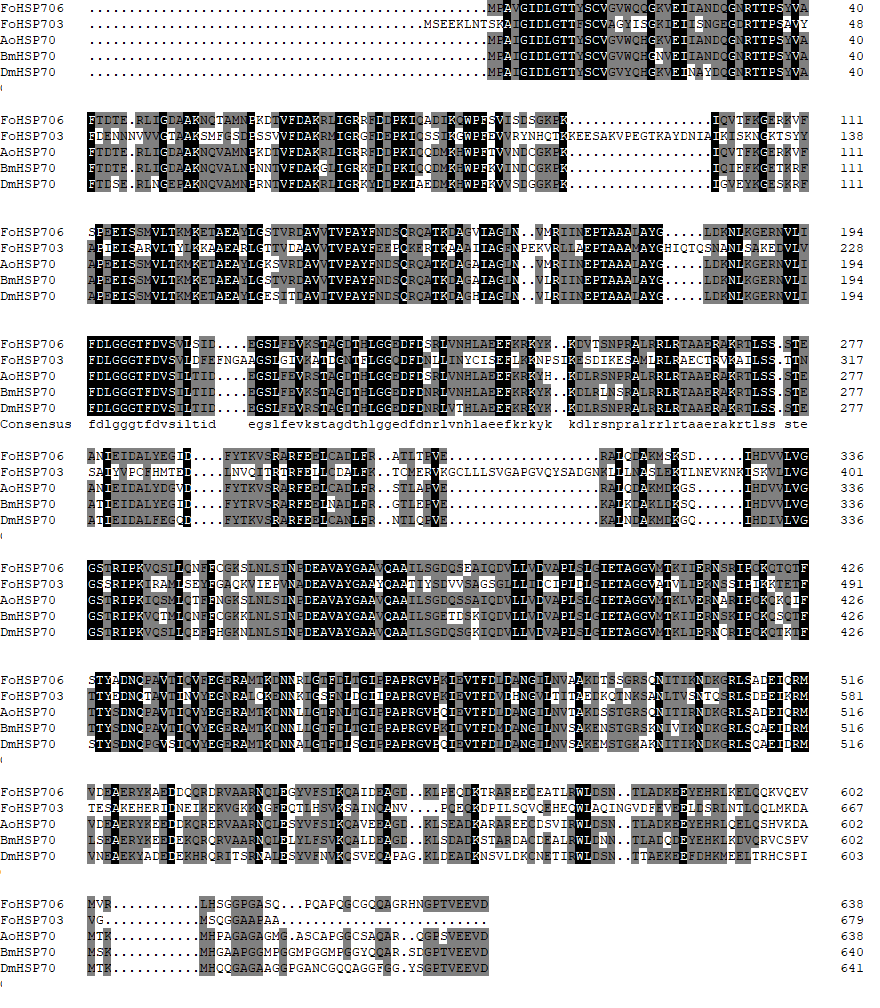

Supplement: Supplemental Information 2 — Multiple sequence alignment of deduced amino acids from FoHSP706 with analogous proteins from other species. [file peerj-07-7687-s002.png]
